# Mainly Dimers and Trimers of Chinese Bayberry Leaves Proanthocyanidins (BLPs) are Utilized by Gut Microbiota: In Vitro Digestion and Fermentation Coupled with Caco-2 Transportation

**DOI:** 10.3390/molecules25010184

**Published:** 2020-01-01

**Authors:** Wenyang Tao, Chaoyang Wei, Shuyu Shen, Mengting Wang, Shiguo Chen, Xingqian Ye, Yanping Cao

**Affiliations:** 1 Beijing Advanced Innovation Center for Food Nutrition and Human Health, Beijing Technology and Business University (BTBU), Beijing 100048, China; wytao@zju.edu.cn; 2 College of Biosystems Engineering and Food Science, National-Local Joint Engineering Laboratory of Intelligent Food Technology and Equipment, Zhejiang Key Laboratory for Agro-Food Processing, Zhejiang Engineering Laboratory of Food Technology and Equipment, Zhejiang University, Hangzhou 310058, China; weichaoyang2012@163.com (C.W.); vera_shensy@163.com (S.S.); mtwang@zju.edu.cn (M.W.); chenshiguo210@163.com (S.C.); psu@zju.edu.cn (X.Y.)

**Keywords:** gastrointestinal digestion, in vitro colonic fermentation, proanthocyanidins absorption, bioavailability

## Abstract

Chinese bayberry leaf proanthocyanidins (BLPs) are Epigallocatechin gallate (EGCG) oligomers or polymers, which have a lot of health-promoting activity. The activity is closely related to their behavior during in vitro digestion, which remains unknown and hinders further investigations. To clarify the changes of BLPs during gastrointestinal digestion, further research is required. For in vitro digestion, including gastric-intestinal digestion, colon fermentation was applied. Caco-2 monolayer transportation was also applied to investigate the behavior of different BLPs with different degrees of polymerization. The trimers and the tetramers were significantly decreased during in vitro gastric-intestinal digestion resulting in a significant increase in the content of dimers. The dimers and trimers were the main compounds utilized by gut microbiota and they were assumed not to degrade through cleavage of the inflavan bond. The monomers and dimers were able to transport through the Caco-2 monolayer at a rate of 10.45% and 6.4%, respectively.

## 1. Introduction

Bayberry (*Myrica rubra* Sieb. et Zucc.), which has been cultured in China for more than 2000 years, belongs to the genus *Myrica* in the family of Myricaceae [1], and is very popular among the locals for its wonderful taste, flavor, and attractive appearance. Leaves from bayberry trees are green throughout the year, and the leaves are pruned twice or more in a year, resulting in a mass of discarded waste [2]. Proanthocyanidins extracted from Chinese bayberry leaves exhibit antioxidant, antitumor, and neuroprotective activity according to previous studies [3,4,5]. The special units of bayberry leaf proanthocyanidins (BLPs), prodelphinidins, were identified in our previous works [2,6]. Compared to proanthocyanidins (Pas) from other resources such as grape seeds, cranberry or apples [7,8,9], BLPs contain a simple but potent unit, Epigallocatechin gallate (EGCG) as the terminal and most of their extension units, with a mean degree of polymerization (mDP) of about 6.5 [10].

Proanthocyanidins (PAs), also known as condensed tannins, are one of the most abundant types of phytochemicals in plants, and are prevalant in in fruits, grains and leaves [11,12,13]. More than 30% of polyphenols consist of PAs in grape [14,15], representing the major part of intake flavonoids, far beyond other phytochemical substances [16]. Due to the differences in subunit composition, PAs can be divided into three categories: procyanidin with the subunit catechin or epicatechin, propelargonidins with afzelechin, and prodelphinidins with gallocatechin or epigallocatechin [17]. The health-promoting potentials of PAs, including antioxidant, antitumor, antivirus, and liver injury protection, were widely investigated in recent decades [12,18,19,20]. Ishihara et al. showed that highly polymeric A-type proanthocyanidins from seed shells prevent the light from damaging the rat retina by inhibiting oxidative stress and apoptotic mechanisms [21]. A hypothesis is proposed that at a low degree of polymerization (DP) proanthocyanidins are good inhibitors of digestive enzymes because of their ability to form specific interactions with enzymes [22]. Grape seed proanthocyanidins inhibit the growth and multiplicity of ultraviolet radiation-induced non-melanoma skin cancer [23]. Daily intake of grape seed proanthocyanidins and/or vitamin C given at the early stage of disease may act in a complementary role in the pharmacological therapy of diabetes and pulmonary vascular dysfunction [24].

However, most studies focus on the health-promoting activities of PAs. Limited studies on PA metabolism and absorption have been published. The health-promoting potentials of PAs depend on their bioavailability, which is quite low in most cases. Proanthocyanidins are reported to be unstable as they degrade during gastric digestion with the impact of gastric acid and enzymes [25]. PAs are also not stable in intestinal digestion, leading to the degradation of PAs into smaller molecules [26]. After gastric-intestinal digestion, PAs are passed into the large intestine and fermented by human gut microbiota into different types of phenyl-γ-valerolactones and phenolic acids [27]. However, Ottaviani et al. and Wiese et al. opposed the proposed acid hydrolysis-driven depolymerization of PAs in the human stomach or the gut microbiome-catalyzed breakdown of PAs into their flavanol subunits [28,29]. These conflicts are raised because of the unclarity of the metabolism pathways of PAs in that the same PAs with different DPs might degrade in different manners. For example, dimer B2 is shown to suffer a A-ring cleavage of the lower unit after a C-ring cleavage of the upper unit, while the monomer does not [30,31,32,33]. The behavior of PAs during in vitro digestion and fermentation should take the DP into consideration since the new metabolites may contribute bioactive effects.

The aim of the present work is to investigate the changes of different DPs of BLPs during in vitro digestion and in vitro fermentation as well as the absorption rate of different DPs of BLPs. For this purpose, the BLPs were applied to in vitro digestion models and in vitro batch-culture fermentation. Also, the BLPs were introduced to Caco-2 monolayers to determine the absorption rate.

## 2. Results and Discussion

### 2.1. PA Content during In-Vitro Digestion

**Figure 1:**
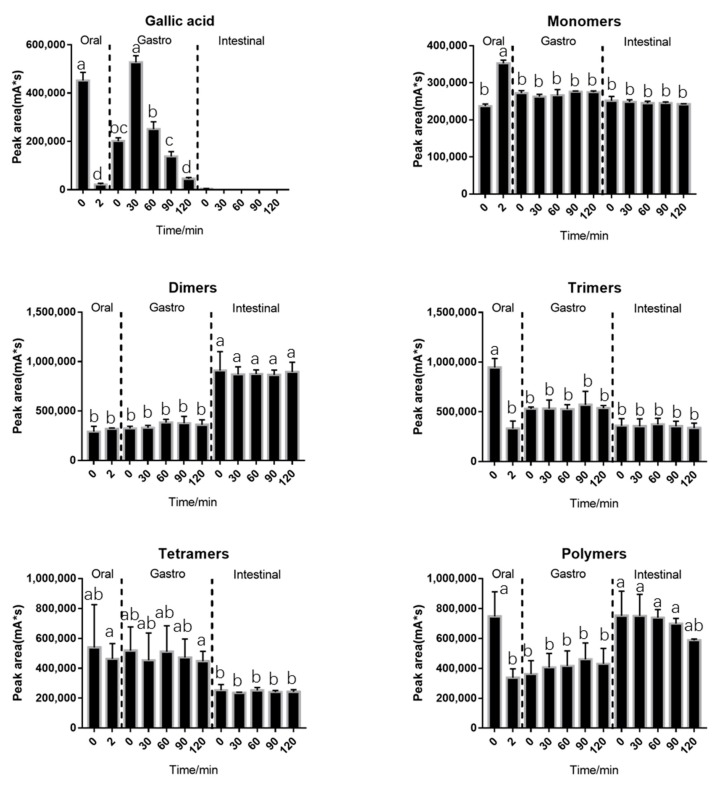
Changes of gallic acid and different degrees of polymerization (DPs) of proanthocyanidins during in vitro digestion. Statistical significance (*p* < 0.05) is expressed by different letters.

**Figure 2:**
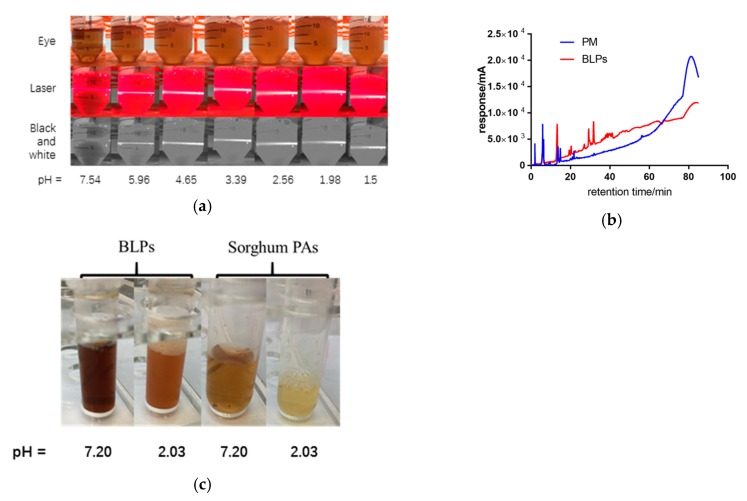
(**a**) BLPs produce sediment under different pH values. (**b**) HPLC of precipitate (PM) and proanthocyanidins (BLPs). (**c**) BLPs and sorghum Proanthocyanidins (Pas) under different pH values.

The behavior of BLPs during in vitro digestion was investigated and shown in Figure 1. Some characteristics of BLPs during in vitro digestion are shown. Gallic acid and trimers were significantly decreased after 2 min of oral digestion. After entering the stomach environment, a significant increase of gallic acid was observed. The gallic acid content reached its maximum level in 30 min, then began to decrease. Gallic acid was not detected during the intestinal digestion except at 0 min. The elevation of gallic acid content during gastric digestion and the reduction during intestinal digestion were consistent with results previously described by Jose et al. [34]. The monomers increased significantly after oral digestion, which was probably due to the degradation of the trimers in oral digestion. On the contrary to Celep et al. who declared that monomers suffer a significant decrease under acidic conditions during in vitro gastric digestion [15], the monomers were rather stable in the results. The dimers showed no significant differences during in vitro digestion, except a significant increase after pH adjustment. The increase of the dimers may be due to the degradation of trimers and tetramers after pH adjustment. There was a significant decrease in trimer content during oral digestion, and some of them might be combined with alfa-amylase as PA is a potent inhibitor of amylase [12], and the other part might be degraded into monomers as mentioned above. After entering the gastric environment, the trimers might be separated from the enzyme due to the acidic environment of the stomach, resulting in an increase in trimer content. The trimers might be unstable under an alkaline environment since they revealed a decrease entering intestinal environment. The tetramers were similar to the trimers. The trimers and tetramers may degrade after entering the intestinal environment or be combined with the pancreas since proteins are known to be precipitate by condensed tannins as the report described [35]. The polymers suffered a significant decrease during oral digestion with their ability to combine with alfa-amylase. On the contrary to the trimers, the polymers did not significantly increase after entering the gastric environment. The trimers and polymers were identified to be significantly reduced during oral digestion, which might be the responsible component that hinders the alfa-amylase [35]. There are two possible explanations for why polymers do not increase during the gastric phase. One is that the polymers contain more hydroxyl groups in one molecular than the trimers which prevents the polymers from separating from amylase in an acid environment [36]. The other explanation is that polymers separate from amylase as trimers but precipitate after entering the acidic environment. Sediment showed up when samples were adjusted to acidic conditions. The data are shown in Figure 2. The polymers continued to decrease during in vitro intestinal digestion, indicating the degradation of polymers.

BLPs produce sediment under acidic conditions along with the decrease of pH value, as shown in Figure 2. The solution becomes more turbid and the laser path in the solution becomes clearer. When the pH value is lower than 2.56 ± 0.05, the light path begins to blur. When the pH value is 1.50 ± 0.05, nearly half of the light path becomes blurred. This indicates that with the decreasing pH value, the content of solid particles in the solution also increased synchronously. The precipitate was collected, dissolved in methanol, and analyzed by HPLC. It was found that the response value increased significantly after 60 min, indicating that most of the precipitates were BLP polymers. Additionally, the precipitate dissolved after adjusting the pH value back to neutral. In a word, the polymers combined with alfa-amylase during oral digestion, separated from amylase but precipitated after entering gastric digestion, and dissolved entering intestinal digestion. The phenomenon does not happen when the subunit is catechin, as shown in Figure 2c. The BLP sample is blurred when the pH value is adjusted to 2, while the sorghum PAs are still clear after adjustment of pH value. The extra hydroxyl group and the galloylation group might contribute to the precipitation of BLPs, which is consistent with the hypothesis that Ma et al. noted in their previous review [36]. Studies like that of Chen et al. noted a decrease in PA content during in vitro digestion and gave credit to acidic-driven degradation or the interaction with PAs and protein which produced precipitate as well [16,37].

This characteristic of BLPs may protect them from the degradation of polymers from acidic conditions, leading to a higher content of PAs transported into the colon and fermented by the gut microbiota.

In summary, the different behavior of PAs with different DPs is shown in the present research. The trimers and polymers might be responsible for the inhibition of amylose according to their dramatic decrease during the oral phase, which is consistent with our previous research [38]. The elevation of the dimers during the intestinal digestion might be a result of degradation of the trimers and tetramers. The polymers were shown to produce precipitate under the acidic conditions of gastric digestion and dissolved in the neutral conditions of intestinal digestion.

### 2.2. PA Content during In-Vitro Fermentation

**Figure 3:**
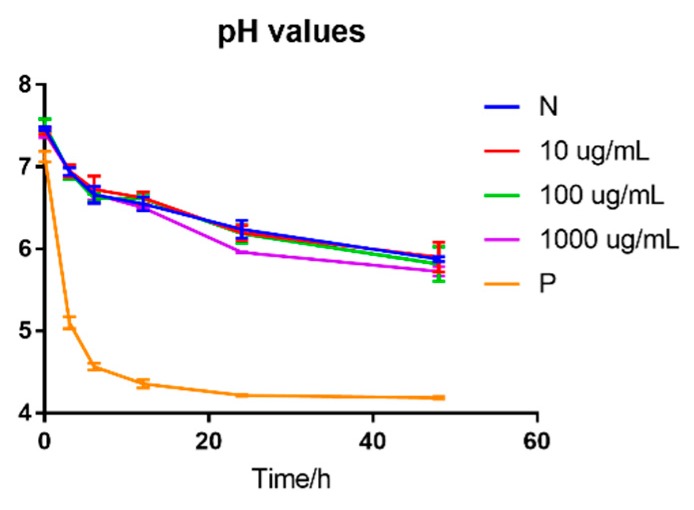
pH values of fermented samples of different concentrations of BLPs. N: blank, negative; P: positive control, samples supplemented with galacto-oligosaccharide.

**Figure 4:**
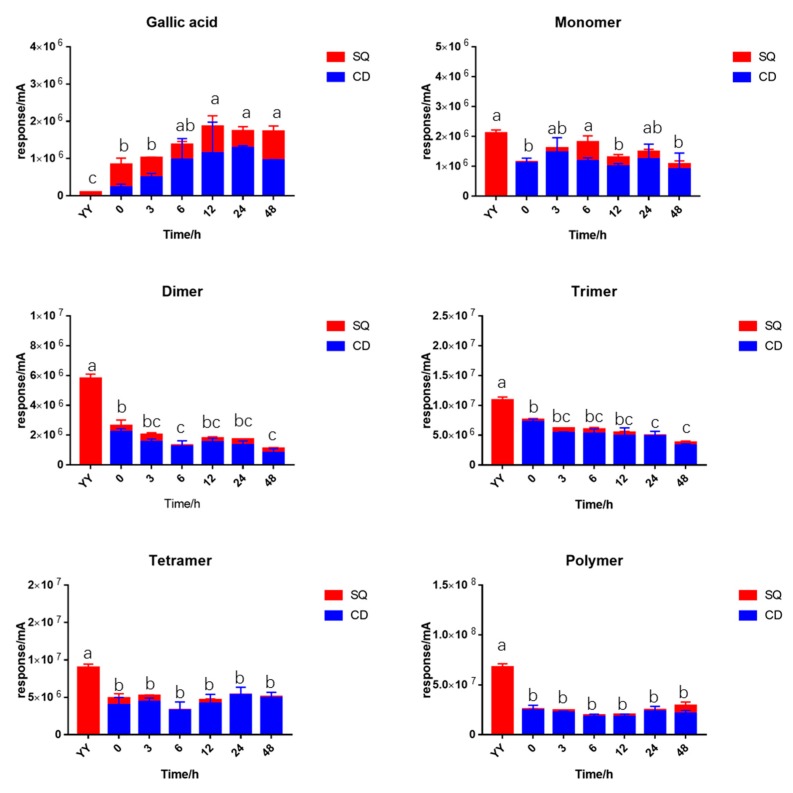
Changes of different degrees of polymers of BLPs (1 mg/mL) during in vitro fermentation. SQ: supernatant; CD: residual; YY; initial sample as 1 mg/mL BLPs in water. Statistical significance (*p* < 0.05) is expressed by different letters.

The pH values of fermented samples of different concentrations of BLPs were shown in Figure 3. No significant difference was found in the pH values of different concentrations from 10 μg/mL to 1000 μg/mL of BLPs during in vitro fermentation, which indicated that BLPs were not a prebiotic for the acid-producing bacteria. The positive control, galacto-oligosaccharide (GOS), revealed a significant decrease after 3 h of fermentation and reached 4.21 ± 0.01. The content of different DPs of BLPs was analyzed and shown in Figure 4. The result was different from Zhou et al. in that the short-chain fatty acid (SCFA) content was shifted by grape seed PAs whose subunits were catechin and epicatechin [39]. The differences may contribute to the different subunits of two samples, that EGCG has a higher molecular weight and more hydroxyl groups [36].

The fermented samples were separated by centrifuge into two parts: the supernatant samples (SQ) and the residual samples (CD). Total proanthocyanidins content (TPAC) or total gallic acid content (TGC) was defined as the summary of SQ and CD samples. The gallic acid was significantly increased compared to the initial sample, indicating the hydrolysis of gallic acid from the EGCG unit during in vitro fermentation. TGC reached a peak after 12 h fermentation and then remained stable. The TPAC of the monomers remained stable during fermentation. The TPAC of the dimers and the trimers revealed a significant decrease throughout fermentation. The tetramers and the polymers remained stable during fermentation. However, they suffered a significant loss after the addition of fecal slurry while TGC significantly increased. The content of PAs in residual is higher than that of the supernatant.

Only the dimers and the trimers were significantly decreased during 48 h fermentation, which raised a hypothesis that the dimers and trimers are the main compounds utilized by gut microbiota. The dimers were reported to degrade into monomers by the cleavage in the inflavan bond [30]. With the degradation of monomers into phenolic acids, the TPAC of monomers remains stable during in vitro fermentation. However, the hypothesis proposed by Wiese et al. and Ottaviani et al. in previous research might be more convincible in that the dimers and trimers were degraded directly to the metabolite without cleavage of the inflavan bond [28,29]. The TPAC of polymers during fermentation was lower than the YY sample thanks to the ability of polyphenols to precipitate with macro molecules such as protein and polysaccharide [40].

### 2.3. Caco-2 Cell Absorption

As shown in Table 1, the absorption rate of BLPs without digestion treatment was measured by the Caco-2 monolayer transport assay. The content of monomers and dimers in the basolateral side was 5.16 ± 2.33 and 8.73 ± 4.95, respectively. The monomers were absorbed at a rate of about 10.45%, and the dimers at 6.4%. The PAs with DPs higher than three were not detected in the basolateral side. The absorption rates were higher than the rat models previously described [41]. In consideration that the molecular weight of a BLP dimer is 914, which is similar to the trimers of procyanidins (866), the result was consistent with Li et al. that the monomers, dimers, and trimers of procyanidins were reported to transverse through 12 kDa cut off cellulose dialysis [42]. The result shows a hypothesis that PAs transverse the Caco-2 monolayer based on molecular weight.

## 3. Material and Methods

### 3.1. Chemicals

Preparation, identification, and the detailed chemical properties of Chinese bayberry leaf proanthocyanidins (BLPs) were described in our previous researches [2,43], detailed information was presented in the supplementary file. Sorghum PAs were extracted the same way as BLPs. Porcine pancreatin and bile salt were purchased from Sigma–Aldrich (St. Louis, MO, USA). Pepsin and α-amylase were purchased from Shanghai Chemical Reagent Company (Shanghai, China). Other reagents in common use were also purchased from Sigma-Aldrich (St. Louis, MO, USA).

### 3.2. In Vitro Digestion

In vitro digestion was carried out as Minekus et al. proposed with slight modifications [44]. The BLPs were dissolved in 8 mL distilled water to form a concentration of 4 mg/mL as a raw sample. Three milliliters of raw samples were stored in −80 °C for future analysis. The three simulated digestion fluids were newly prepared before assay with the enzymes was added.

For oral digestion, 5 mL of simulated saliva fluid that was composed of amylase enzyme (75 U/mL) was added and incubated in a shaking water bath for 2 min at 37 °C. Three milliliters of the oral sample was collected after oral digestion.

After oral digestion, 7 mL simulated gastro fluid that was composed of pepsin enzyme (2000 U/mL) was added to the mixture and the pH was adjusted to 3.0 with 6 mol/L HCl to mimic the acidic condition in the gastric. The mixture was incubated for 2 h in a shaking water bath at 37 °C. Samples were collected every 30 min at a volume of 1 mL including the sample after pH adjustment and an extra 2 mL sample of 120 min.

For intestinal digestion, 8 mL simulated intestinal fluid that was composed of porcine pancreatin (100 U/mL based on trypsin activity) and bile salt were added to the mixture and the pH was adjusted to 7 with 1 mol/L NaOH. The mixture was incubated for 2 h at 37 °C. Samples were collected every 30 min at a volume of 1 mL.

### 3.3. In Vitro Fermentation

The interactions between BLPs and human intestinal microbiota were investigated by in vitro fermentation according to the method described in previous research with some modifications [39]. The protocol number of ethical committee approbation for the obtention of fecal samples is ZJU-BEFS-2018001. Briefly, fecal samples were obtained from five healthy volunteers (two females and three males, age 25–30, who lived in Hangzhou, China for more than a year) without antibiotic treatment over the preceding 6-month period and without a gastrointestinal disorder. The volunteers were mentally and physically able to participate in the study. In addition, the experiments were performed in compliance with the relevant laws and institutional guidelines. The fecal slurries were prepared by mixing fresh fecal samples with autoclaved phosphate buffered saline (PBS, 0.1 mol/L, pH 7.2) to yield 10% (*w*/*v*) suspensions. The sample was mixed with autoclaved basal nutrient growth medium (10% *v*/*v*) in an anaerobic incubator of YQX-II (Yuejin Medical Optical Instruments Factory, Shanghai, China). The basal nutrient medium (pH 7.0) contained (per liter) 2 g peptone, 2 g yeast extract, 0.1 g NaCl, 0.04 g K_2_HPO_4_, 0.04 g KH_2_PO_4_, 0.01 g MgSO_4_·7H_2_O, 0.01 g CaCl_2_·6H_2_O 0, 2 g NaHCO_3_, 0.02 g hemin, 0.5 g cysteine HCl, 0.5 g bile salts, 1 mg resazurin, 2 mL Tween 80, 10 μL vitamin K1, and distilled water. Fermentation was initiated by adding 5 mL of the fecal slurry to 45 mL of culture medium containing different concentrations (10 μg/mL, 100 μg/mL, 1000 μg/mL) of BLPs as samples and galacto-oligosaccharide (GOS) as a positive control and incubated at 37 °C in the anaerobic incubator. In the present study, the culture medium without the addition of any compound used as a negative control was prepared under the same conditions. Samples (5 mL) were taken from the inoculums at 0, 3, 6, 12, 24, and 48 h for further analysis. The fermented samples were separated into two parts, the supernatant samples (SQ) and the residual samples (CD), by centrifuging at 5000 rpm for 3 min. The CD samples were extracted with 70% acetone to obtain the combined PAs. All samples were stored in −80 °C refrigerator and all experiments were repeated three times.

### 3.4. Caco-2 Absorption Assay

#### 3.4.1. Cell

Caco-2 cells (Cat. No. TCHu146), the epithelial cells from human colorectal adenocarcinoma, were purchased from the Type Culture Collection of the Chinese Academy of Sciences (Shanghai, China).

#### 3.4.2. Cell Culture

The Caco-2 cells were grown in dulbecco’s modified eagle medium (DMEM) containing 10% fetal bovine serum, 1% nonessential amino acid (NEAA), 100 units/mL penicillin, 100 μg/mL streptomycin, and 4 mmol/L l-glutamine at 37 °C in an atmosphere of 5% CO_2_ and 90% relative humidity. Stock cultures were grown in 25 cm^2^ tissue culture flasks and were split at 80% to 90% confluency using 0.25% trypsin and 0.02% Ethylenediaminetetraacetic acid (EDTA) solution.

#### 3.4.3. Thiazolyl Blue Tetrazolium Bromide (MTT) Assay

Passages 30–45 of Caco-2 cells were used for MTT assay and performed in triplicates. The BLPs were dissolved with culture medium in gradient concentrations. Caco-2 cells were cultured in 96-well plates (Corning, New York, USA) at 5 × 10^4^ cells/well in 100 μL of the medium in a humidified atmosphere with 5% CO_2_ at 37 °C. After 24 h incubation, the culture medium was replaced with testing samples and culture medium (negative control) then exposed for 24 h. The following procedures were performed as described by Zhang et al. [45]. The MTT reagent was added to 96-well plate and incubated for 2 h. The optical density (OD) of formazan was measured at 490 nm on a microplate reader (ThermoFisher Scientific, Waltham, USA). Wells without cells were used as blank control. A concentration with a viability of more than 90% is considered as non-toxic to cells.

#### 3.4.4. Caco-2 Absorption

Caco-2 absorption assay was performed according to Ding et al. with slight modifications [46]. Cells were seeded at 1 × 10^5^ cells/cm^2^ onto permeable inserts (1.0 µm, Polyethylene terephthalate (PET), for 6-well plates, Millipore, Kankakee, IL, USA) in 6-well plates. The culture medium in inserts and plates was replaced every two days for the first week after seeding and replaced daily afterward. The integrity of the cell monolayers was examined by measuring the transepithelial electrical resistance (TEER) with Millicell^®^ ERS-2 Volt-Ohm meter (Millipore, Kankakee, IL, USA). The cells were used for the transport studies approximately 21 days after seeding, with a TEER value above 250  Ω (4.67 mm^2^) in Hank’s Balanced Salt Solution (HBSS) during the studies. Testing samples: 1500 μL of BLPs dissolved in HBSS were loaded on apical sides (AL), and 2600 μL of HBSS were added to the basolateral (BL) side. Samples were collected after 2 h incubation from the BL side. All incubations were performed in triplicates.

### 3.5. Quantification of PAs

HPLC analysis was performed as previously described by Holland et al. with slight modifications for BLPs [47]. Samples were filtered with a 0.22 μm membrane before HPLC analysis. The analysis was performed on a Waters e2695 Separations Module equipped with a Waters 2489 UV/Vis Detector and a HILIC column (250 × 4.6 mm, 5 μm). The mobile phase A was acetonitrile consisting of 0.1% acetic acid, the mobile phase B was methanol consisting of 0.1% acetic acid and 3% water, the flow rate was set to 0.5 mL/min, the column temperature was set to 25 °C. Gradient elution was performed as follows: 0–3 min, 7% B; 3–15 min, 7–23% B; 15–70 min, 23–65% B; 70–85 min, 65%–100% B. The injection volume was 10 μL. The detection wavelength was set to 280 nm.

### 3.6. Statistical Analysis

Data are presented as the mean ± standard deviation of three independent experiments. Statistical significance was determined with software SPSS. *p* < 0.05 was considered to indicate a statistically significant difference.

## 4. Conclusions

The behavior of bayberry leaf proanthocyanidins (BLPs) with different degrees of polymers (DPs) was investigated in the present research. The subunits of BLPs, EGCG, were partly hydrolyzed and produced gallic acid during in vitro digestion. The trimers and the tetramers were degraded into the dimers during in vitro digestion. The polymers produced precipitate under the acidic condition of the gastric digestion and dissolved under the neutral condition of the intestinal digestion. The phenomenon was not discovered in sorghum proanthocyanidins whose subunits are catechin and epicatechin, indicating that different subunits may alter not only the activity of PAs but also their absorption and metabolism.

The in vitro batch fermentation model was introduced to different concentrations of BLPs. The pH values were not influenced by BLPs, indicating that BLPs were not a potent prebiotic for acid-producing bacteria. The dimers and the trimers were the only two components that significantly decreased during in vitro fermentation. With the stability of the monomers, the proposed hypothesis that PAs degrade directly into their metabolites was proved. The Caco-2 absorption assay was applied in the research. The results showed that proanthocyanidins might transverse Caco-2 monolayer based on molecular weight.

## Figures and Tables

**Table 1:** The absorption rate of BLPs by Caco-2 monolayer.

	AL	BL	Ratio
Monomers	49.37 ± 9.15	5.16 ± 2.33	10.45%
Dimers	135.69 ± 13.48	8.73 ± 4.95	6.4%

AL: apical side; BL: basolateral side; the content of monomers and dimers are expressed as μg Epigallocatechin gallate (EGCG) equivalate. Ratio: (BL/AL%).

## Supplementary Materials

The following are available online https://www.mdpi.com/1420-3049/25/1/184/s1, Figure S1: Reverse phase HPLC chromatograms (detected at 280 nm) of reaction products of (a) CBLPs, (b) SPBLPs and (c) UDBLPs with the presence of excess phloroglucinol. Peak 1: ascorbic acid; peak 2: phloroglucinol; peak 3: EGCG-ph; peak 4: EGCG. Table S1: Mean degree of polymerization (mDP), content of proanthocyanidins (PAs) and total phenolic content (TPC) of different varieties of Chinese bayberry leaves proanthocyanidins (BLPs), Table S2: Fractions from BLPs and their identification from normal-phase preparativie HPLC-ESI/MS and reverse-phase HPLC-ESI/MS.
