# Supplementary material for: Mainly Dimers and Trimers of Chinese Bayberry Leaves Proanthocyanidins (BLPs) are Utilized by Gut Microbiota: In Vitro Digestion and Fermentation Coupled with Caco-2 Transportation
Source: Molecules. 2020 Jan 1;25(1):184. doi: 10.3390/molecules25010184 (PMC6982776; doi:10.3390/molecules25010184)
Supplement: Supplementary file 1 [file molecules-25-00184-s001.pdf]

SUPPLEMENTARY MATERIALS

1. Methods and Materials

1.1. Extraction, Purification, and Degradation of Proanthocyanidins (PAs)

Extraction and purification of PAs from the leaves were based on a previous method (Fu, Qiao, Cao, Zhou, Liu, and Ye, 2014; Yang, Ye, Liu, Chen, Zhang, Shen, et al., 2011). Briefly, the leaves were dried in the oven at 40 °C for 12 h and ground into powder with the particle size around 150 μm. After the leaf powder (4 kg) was extracted with 70% acetone (40 L), the aqueous phase was obtained and washed with hexane and dichloromethane. Then, the organic solvent was evaporated by rotary evaporation and the aqueous phase was lyophilized to dryness to obtain the crude bayberry leaves proanthocyanidins extract (CBLPs). CBLPs were then purified by a HPD-500 column to remove sugar with ethanol as an elution solvent. The eluate was evaporated to dryness yielding a brown powder, which was labeled as resin purified BLPs (RPBLPs). Afterwards, RPBLPs were purified by a Sephadex LH-20 (300 mm × 30 mm i.d.) column. Then, 90% methanol was used to elute most of the flavonoids and after 50% acetone was applied to elute most PAs and collected. The collection was dried by rotary evaporation under vacuum to remove organic solvent and then lyophilized to dryness to obtain SPBLPs. In order to prepare PAs with a low mDP, 50 mg of SPBLPs were redissolved in 25 mL of ethanol and the mixture was depolymerized by ultrasound treatments with a probe ultrasonic processor (JY92-IIDN, Ningbo Scientz Biotechnology Co., Ningbo, China) under the following conditions: temperature of 15 °C; pulsed mode of 2 s on and 1 s off; ultrasound intensity of 300 W/cm^2; and treatment time of 15 min. Ethanol was rotary-evaporated from the mixture under

vacuum, and then, the solution was lyophilized to dryness and labeled as ultrasonic depolymerized bayberry leaf proanthocyanidins (UDBLPs).

1.2. Determination of Total Phenolics and PAs

The total phenolic content (TPC) was determined using the Folin–Ciocalteu assay (Bao et al., 2005). In brief, 400 μL of the prepared sample solution was added to 1 mL of 1 M Folin–Ciocalteu reagent. The sample was diluted to 2 mL with distilled deionized water and shaken completely. The mixture solution was maintained in the dark for 5 min at room temperature. Then, 5 mL of 5% Na_2CO_3 (w/v) was added to the mixture, which stood for 60 min at room temperature. Finally, the absorbance of the mixture solution was measured at 765 nm. The results were expressed as mg of gallic acid equivalents per gram dried weight (DW).

The PA content was determined according to a modified vanillin assay (Sun, Leandro, Ricardo da Silva, and Spranger, 1998). Briefly, 2.5 mL of 1% (w/v) vanillin in methanol and then 2.5 mL of 20% (v/v) H_2SO_4 in methanol were added to 1 mL of the prepared sample solution to perform the vanillin reaction. Then the mixture solution was kept in a 30 °C water bath for 15 min to undergo the vanillin reaction. Methanol was used as the blank. The absorbance was measured at 500 nm and compared to the prepared blank. The content of PAs was calculated based on the standard curve of EGCG.

1.3. Liquid Chromatography Analysis of Mean Degree of Polymerization (mDP) of BLPs

The mDP of the PAs was determined using acid catalysis in the presence of phloroglucinol based on our previous procedures (Yang, et al., 2011). In brief, the terminal flavan-3-ols units were cleaved by acid catalysis in the presence of phloroglucinol, while the extension units were released as phloroglucinol derivatives (Kennedy and Jones, 2001). Then 5 mg of PA samples were dissolved in 1 mL of a freshly prepared methanol solution containing 0.2 N HCl, 50 g/L phloroglucinol, and 10 g/L ascorbic acid. The mixture solution was kept at 60 °C for 1 h to perform the reaction. Afterwards, 1 mL of 200 mM of sodium acetate was added to stop the reaction. The reaction mixture was then filtered through a 0.45-μm membrane and analyzed by a reverse-phase HPLC-DAD platform. Separation was performed on a Zorbax SB-C18 (Agilent, Santa Clara, CA, USA) column (250 × 4.6 mm, 5 μm) at 35 °C and was detected at 280 nm. Mobile phase A was 0.1% formic acid, and mobile phase B was methanol (flow rate: 1 mL/min). The gradient elution profile was as follows: 5% B (0–10 min), 5%–20% B (10–20 min), 20%–40% B (2030 min), and 40%–90% B (35–37 min).

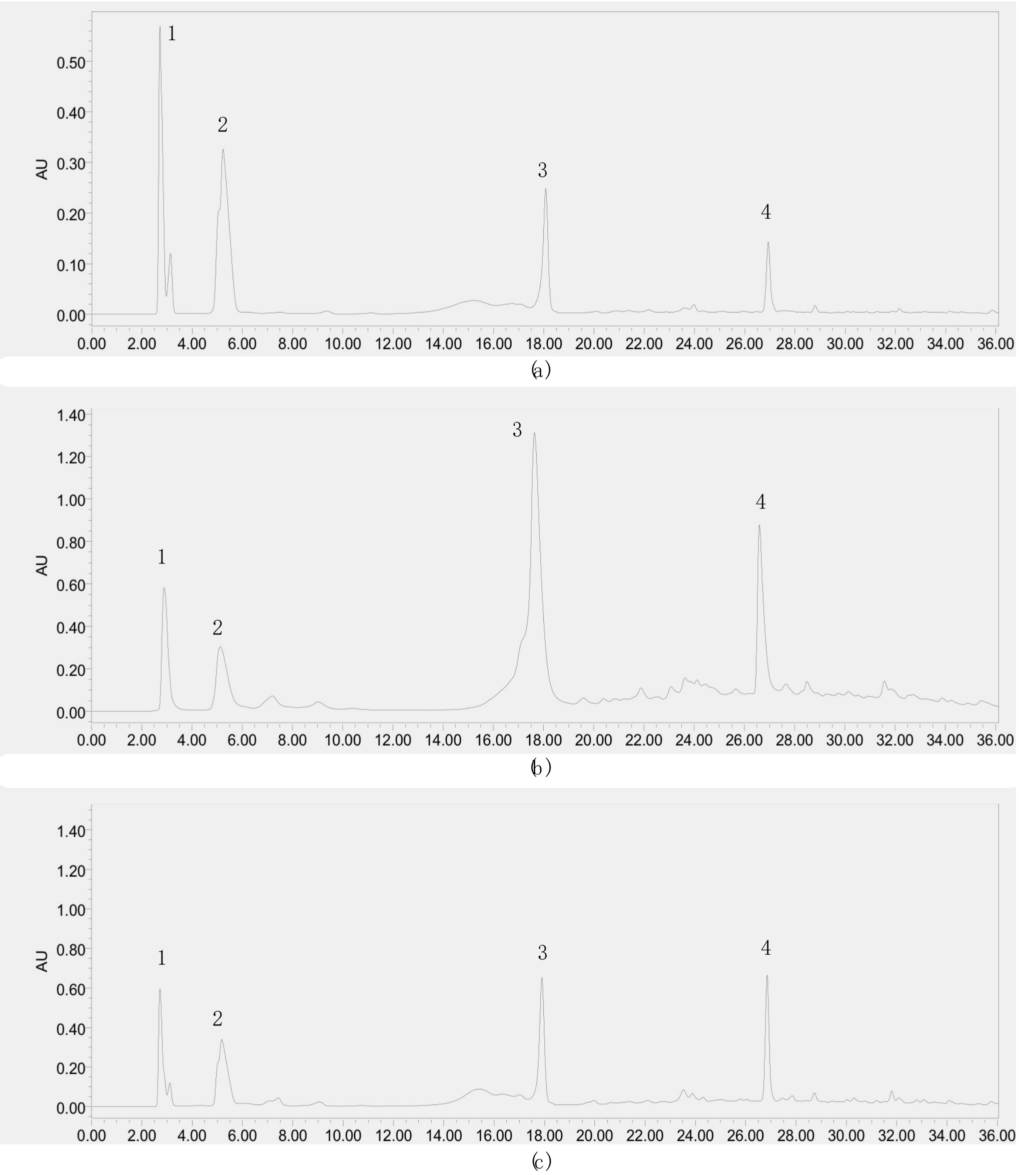

Figure S1. Reverse phase HPLC chromatograms (detected at 280 nm) of reaction products of (**a**) CBLPs, (**b**) SPBLPs, and (**c**) UDBLPs with the presence of excess phloroglucinol. Peak 1: ascorbic acid; peak 2: phloroglucinol; peak 3: EGCG-ph; peak 4: EGCG.

Table S1. Mean degree of polymerization (mDP), content of proanthocyanidins (PAs), and total phenolic content (TPC) of different varieties of Chinese bayberry leaves proanthocyanidins

(BLPs).

Varieties	TPC	PAs	mDP	Extension units	Terminal units
		(%)		EGCG-ph (%)	EGCG (%)
CBLPs	242.81 ± 0.55[c]	36.3 ± 1.6[c]	6.1 ± 0.3[b]	11.68	4.65
UDBLPs	353.82 ± 1.37[b]	80.8 ± 0.5[b]	3.6 ± 0.3[c]	11.57	8.54
SPBLPs	378.28 ± 0.97[a]	85.7 ± 1.1[a]	7.3 ± 0.1[a]	28.78	9.75

[1] Data are expressed as mean values ± SD (n = 3). Values with no letters in common with each column are significantly different ($p < 0.05$). [2] Total phenolics were expressed as mg of gallic acid equivalents per gram DW. [3] mDP, mean degree of polymerization, mDP = ((extension units) + (terminal units))/(terminal units).

1.4. Characterization of BLPs

The characterization of Chinese bayberry leaves proanthocyanidins (BLPs) and its structural elucidation have been previously reported by our group (Fu, Qiao, Cao, Zhou, Liu, and Ye, 2014; Yang, Ye, Liu, Chen, Zhang, Shen, et al., 2011). Yang et al. found that the structure of BLPs was special and of the prodelphinidin type. By the acid catalysis with excess phloroglucinol, epigallocatechin-3-O-gallate (EGCG) and traces of epigallocatechin (EGC) were detected as the extension units, but only EGCG was present in the terminal units. BLPs exhibited a 2,3-cis configuration, and more than 98% of them were galloylated. Afterwards, Fu et al. further supported the results of Yang et al. and the fractions from BLPs and their identification from normal-phrase HPLC-ESI/MS and reverse-phase-ESI/MS were also reported (Table S2). The major components of BLPs were: 5.4% monomers, 10.9% dimers, 13%

trimers, 47.8% tetramers, 22.9% polymers, and other polyphenols.

Table S2. Fractions from BLPs and their identification from normal-phase preparative HPLC-ESI/MS and reverse-phase HPLC-ESI/MS (Fu, Qiao, Cao, Zhou, Liu, and Ye, 2014).

Fractions[a]	Yield[b] (mg/200 mg)	MW[c]	Tentative identification[d]
1	10.8 ± 0.3	616	myricetin deoxyhexoside-gallate
2	4.6 ± 0.1	744, 882	(E)GC + (E)CG, 2(E)CG, 2(E)C + (E)GC
3	8.6 ± 0.3	762	(E)GC + (E)GCG
4	8.6 ± 0.4	914	2(E)GCG
5	4.3 ± 0.2	1066	2(E)GC + (E)GCG
6	8.7 ± 0.2	1218	(E)GC + 2(E)GCG
7	13.0 ± 1.5	1371	3(E)GCG, 3(E)GC + (E)GCG
8	19.6 ± 0.6	1523	2(E)GC + 2(E)GCG
9	32.2 ± 1.6	1675	(E)GC + 3(E)GCG
10	43.8 ± 1.0	1827	3(E)CG + (E)GCG, 4(E)GCG

[a] Fractions and their identification from normal-phase preparative HPLC-ESI/MS and reverse-phase HPLC-ESI/MS.

[b] Yield of one injection of preparative HPLC, that is, mg per 200 mg BLPs.

[c] MW, molecular weight.

[d] Tentative identification, (E)GC, (E)GCG, (E)CG are abbreviations for (epi)gallocatechin, (epi)gallocatechin-3-O-gallate, (epi)catechin-3-*O*-gallate.

References:

Bao, J., Cai, Y., Sun, M., Wang, G., and Corke, H. (2005). Anthocyanins, flavonols, and free radical scavenging activity of Chinese bayberry (Myrica rubra) extracts and their color properties and stability. Journal of Agricultural and Food Chemistry, 53(6), 2327-2332.

Fu, Y., Qiao, L., Cao, Y., Zhou, X., Liu, Y., and Ye, X. (2014). Structural elucidation and antioxidant activities of proanthocyanidins from Chinese bayberry (Myrica rubra Sieb. et Zucc.) leaves. PLoS One, 9(5), e96162.

Kennedy, J. A., and Jones, G. P. (2001). Analysis of proanthocyanidin cleavage products following acid-catalysis in the presence of excess phloroglucinol. Journal of Agricultural and Food Chemistry, 49(4), 1740-1746.

Sun, B., Leandro, C., Ricardo da Silva, J. M., and Spranger, I. (1998). Separation of Grape and Wine Proanthocyanidins According to Their Degree of Polymerization. Journal of Agricultural and Food Chemistry, 46(4), 1390-1396.

Yang, H., Ye, X., Liu, D., Chen, J., Zhang, J., Shen, Y., and Yu, D. (2011). Characterization of unusual proanthocyanidins in leaves of bayberry (Myrica rubra Sieb. et Zucc.). Journal of Agricultural and Food Chemistry, 59(5), 1622-1629.
